# Vitamin D Enhances Radiosensitivity of Colorectal Cancer by Reversing Epithelial-Mesenchymal Transition

**DOI:** 10.3389/fcell.2021.684855

**Published:** 2021-08-04

**Authors:** Xinyue Yu, Qian Wang, Baocai Liu, Ning Zhang, Guanghui Cheng

**Affiliations:** Department of Radiation Oncology, China-Japan Union Hospital of Jilin University, Changchun, China

**Keywords:** vitamin D, radiosensitivity, colorectal cancer, epithelial-mesenchymal transition, radiotherapy

## Abstract

Colorectal cancer (CRC) is often resistant to conventional therapies. Previous studies have reported the anticancer effects of vitamin D in several cancers, its role in radiotherapy (RT) remains unknown. We found that 1α, 25-dihydroxyvitamin D_3_ (VD_3_), the biologically active form of vitamin D, had antitumor effect on CRC and sensitized CRC cells to ionizing radiation (IR). VD_3_ demonstrated synergistic effect in combination with IR, which were detected by colony formation and cell proliferation assay. Radiosensitivity restoration induced by VD_3_ was associated with a series of phenotypes, including apoptosis, autophagy, and epithelial-mesenchymal transition (EMT). Using proteomics, “regulation of cell migration” and “cadherin” were found to be obviously enriched GO terms. Moreover, cystatin D and plasminogen activator inhibitor-1 (PAI-1), the differentially expressed proteins, were associated with EMT. Next, we confirmed the contributions of these two genes in enhancing IR sensitivity of CRC cells upon inhibition of EMT. As determined by proteomics, the mechanism underlying such sensitivity involved partially block of JAK/STAT3 signaling pathway. Furthermore, VD_3_ also elicited sensitization to RT in xenograft CRC models without additional toxicity. Our study revealed that VD_3_ was able to act in synergy with IR both *in vitro* and *in vivo* and could also confer radiosensitivity by regulating EMT, thereby providing a novel insight for elevating the efficacy of therapeutic regimens.

## Introduction

Colorectal cancer (CRC) is reported to be the second and third most common cancer in women and men, respectively, with a high incidence and mortality (Dekker et al., 2019). Despite several treatment strategies, the overall prognosis of advanced CRC remains dismal. Radiotherapy (RT) plays a significant role in the survival of CRC patients. However, intrinsic and acquired radioresistance are the major causes of subsequent tumor recurrence and metastasis. Previous studies have reported various types of cell death involved in radiation-induced resistance (Kim et al., 2015). Therefore, elucidating the molecular mechanisms underlying such resistance and discovering potential sensitizers will aid in promoting therapeutic efficiency.

Up to now, there are several reports exploring the association between vitamin D and cancer risk, especially in CRC (Urashima et al., 2019; Yonaga et al., 2019; Amrein et al., 2020). 1α,25-dihydroxyvitamin D_3_ (VD_3_, also known as calcitriol), activated by binding to nuclear vitamin D receptor (VDR) in the genomic way, displays its wide-ranging effects on a variety of cancers (Jeon and Shin, 2018; Carlberg and Munoz, 2020). Dunlap et al. (2003) demonstrated that vitamin D increased the apoptotic rate in irradiated prostate cancer cells. Additionally, Demasters et al. (2006) reported that EB1089 (vitamin D analog) induced cytotoxic autophagic cell death in breast cancer. In NSCLC, EB1089 elicited cytostatic autophagy, which promoted tumor suppression with no alteration of autophagy extent (Sharma et al., 2014). Such experiments support the notion that vitamin D acts pleiotropically and in various combinations to exert synergistic antitumor effects in different tumors, but the cell-intrinsic signals that sustain the reversal of resistance to therapeutics remain elusive. Consolidating the connection between vitamin D and conventional treatment strategies and unraveling the precise signaling pathways that lead to the restoration of radiosensitivity will provide important implications for clinical CRC therapy.

Epithelial-mesenchymal transition (EMT), which is orchestrated by EMT-inducing transcription factors (EMT-TFs), is involved in therapeutic resistance by generating a series of phenotypic states (Dongre and Weinberg, 2019). Vitamin D has been found to participate in the inhibition of tumor migration and invasion in various cancers. Xu et al. (2020) reported that calcitriol led to suppression of several signaling pathways associated with EMT in renal cell carcinoma. MART-10, another vitamin D analog, was testified to prohibit cell migration in anaplastic thyroid cancer (Chiang et al., 2015). In our study, proteomics data shed light on how vitamin D was able to give rise to sensitivity of CRC to radiation by regulating a variety of phenotypes, and there was no study for providing mechanistic insight into the vitamin D-mediated reversal of EMT in combination with IR. This study aimed to unravel how VD_3_ successfully alleviated therapeutic resistance as well as the mechanism underlying such sensitization.

## Materials and Methods

### Reagents and Cell Lines

CRC cell lines (SW480 and HCT116) were purchased from cell bank in Beijing, China. Cells were cultured in RPMI-1640 or 5A supplemented with 10% FBS and 1% penicillin/streptomycin, and incubated at 37°C humidified atmosphere with 5% CO_2_. VD_3_ and chloroquine (CQ) were purchased from Sigma. Antibodies against GAPDH, cleaved-caspase3, p21, γH2AX, p62, E-cadherin, Claudin-1, Snail, and β-catenin were from Cell Signaling Technology. Antibodies against Bcl-2, Bax, PAI-1, cystatin D, Stat3, p-Stat3, Smad3 were from Santa Cruz Biotechnology. Antibody against LC3B was from Sigma, and TGF-β was from Abcam.

### Cell Viability Analysis

A total of 3 × 10^3^ cells/well were seeded in 96-well plates and the cultured CRC cells were subsequently treated with ethanol (Control), VD_3_, IR, and IR plus VD_3_. Cell viability was analyzed by the CCK8 assay (Beyotime, China). Cells were incubated with 10 μL of CCK-8 solution at 37°C for 2 h. The absorbance of mixture was measured at 450 nm with BioTak Elx808.

### IR Scheme

Cells were seeded in 6-cm dishes and exposed to 6 MV X-ray at the rate of 2 Gy/min (linear accelerator, Elketa/Sweden). A 1.5-cm-thick bolus was used to correct the distribution of IR.

### Colony Formation Assay

Cells with or without VD_3_ treatment were exposed to IR doses of 0, 2, 4, 6, and 8 Gy, followed by incubation for 2 weeks. Cells were then fixed in methanol and stained with 0.5% crystal violet. Single colonies consisting of more than 50 cells were scored. The surviving fraction (SF) was calculated using the following formula: Plating efficiency (PE) = number of colonies formed/number of cells seeded; SF = number of colonies formed after IR/number of cells seeded × PE. Survival curves were fitted using the single-hit multitarget model formula: S = 1-(1-e^–D/D0^)^N^.

### Apoptosis Assay

Cells were seeded at 4 × 10^5^ cells/well in 6-well plates and treated with VD_3_ or IR. After 48 h, cells were harvested and measured by the Annexin V-fluorescein isothiocyanate (FITC) kit (Sungenebiotech, China) according to the manufacturer’s instructions, followed by flow cytometric analysis (Becton CYTOMINCS FC500, United States).

### Cell Cycle Assay

Approximately 3 × 10^5^ cells/well were incubated with the indicated treatment for 48 h. Cells were harvested and fixed with cold 75% ethanol overnight at 4°C, and then stained with propidium iodide (Sigma, United States) and RNase staining buffer for 30 min in the dark. Flow cytometer was subsequently used to measure the DNA content and the obtained data were analyzed with Multicycle AV DNA software.

### Senescence-Associated β-Galactosidase Assays

Cells with the indicated treatment were incubated for 48 h. Cell senescence was detected by a staining kit (Cell Signaling Technology, United States) according to the manufacturer’s instructions. The blue senescent cells were counted under light microscopy.

### Western Blot

Proteins were lysed by RIPA buffer (Beyotime, China) with protease/phosphatase inhibitor cocktail, and the concentration was determined by the BCA Protein Assay Kit (Beyotime, China). Proteins were electrophoretically separated on SDS-PAGE and transferred to a nitrocellulose membrane (Amersham, Germany). The membrane was blocked with skim milk for 1 h at room temperature and incubated with primary antibodies overnight at 4°C. Proteins of interest were incubated with appropriate IRDyeTM 800/700CW secondary antibodies and finally imaged using the Odyssey system (LI-COR Odyssey, United States).

### Transmission Electron Microscopy

Treated SW480 cells were fixed in 4% glutaraldehyde solution, then post-fixed in 1% osmic acid, dehydrated, embedded, sectioned by LEICA EM UC7 ultramicrotome, and stained with uranyl acetate and lead citrate. Ultrathin sections were examined with a FEI TECNAI SPIRIT electron microscope.

### Immunofluorescence Staining

γH2AX foci were detected to evaluate DNA damage. LC3B puncta were employed to monitor the autophagic flux. SW480 cells were grown in the confocal cell culture dish and treated with experimental conditions as indicated. Cells were washed with PBS three times and fixed with 4% paraformaldehyde for 20 min at room temperature. After washing with PBS three times, cells were permeabilized with 0.2% Triton X-100, blocked with 5% BSA in PBS, and then incubated with the anti-LC3B or anti-γH2AX antibodies (all 1:100) at 4°C overnight. After incubating with the secondary antibody for 1 h, samples were counterstained with DAPI (Abcam, United Kingdom) for 5 min. Finally, images were captured with Olympus FV1000 confocal laser scanning microscope.

### Wound Healing Assay

SW480 cells were seeded in 6-well plates and grown overnight to 90% confluency. Then the cell monolayers were scratched with 200 μL pipette tips to form a uniform wound. The plates were washed with PBS and cultured in 1% FBS medium. Cells were photographed at indicated time points using the Olympus IX51 inverted microscope. The wound area was calculated by Image J software with the wound area at 0 h set at 100%.

### Transwell Migration and Invasion Assays

Cell migration and invasion were evaluated using a 24-well plate (Costar Corning, United States). For the migration assay, a suspension of 7 × 10^4^ cells in 200 μL complete medium was added in the upper compartment. After the indicated treatment, RPMI-1640 containing 20% FBS (500 μL) was added to the lower chamber, and complete medium in the upper chamber was replaced with serum-free medium. After incubation for 48 h, the membranes were fixed with methanol for 15 min and stained with crystal violet solution for 20 min. Then cells on the upper filter surface were wiped away with a cotton swab and remaining cells on the opposite side of the filter were counted at 10 × magnification. For the invasion assay, 50 μL matrigel (BD Biosciences) basement membrane (diluted 1:10) was precoated in the upper chamber and incubated at 37°C for 4 h.

### Label-Free Quantitative Proteomics

A label-free quantitative proteomics approach to quantify the dynamic changes in the whole proteome of cell lines was utilized by PTM Biolabs. SW480 cells (1 × 10^7^) were treated with or without VD_3_ and IR for 24 h. Cell lysates were separated on SDS-PAGE and gel samples were collected. Four treatment groups were included in this analysis. Briefly, the workflow encompassed protein extraction, trypsin digestion, labeling, HPLC fractionation, liquid chromatography-tandem mass spectrometry (LC-MS/MS), database search, and bioinformatic analysis. The fold-change cutoff was set when proteins with quantitative ratios above 1.5 or below 1/1.5 and *p*-value < 0.05 were deemed significant.

### RNAi Treatment

SW480 cells transfected with the indicated small interfering RNAs (siRNAs) using GP-transfect-Mate for 48 h were harvested. The sequences of small interfering RNA (siRNA) were as follows:

SiPAI-1#1, 5′-GCCACUGGAAAGGCAACAUTT-3′
SiPAI-1#2, 5′-GCUGACUUCACGAGUCUUUTT-3′
SiCST5#1, 5′-CCAUGCCACAGACCUCAAUTT-3′
SiCST5#2, 5′-GCGAGUACAACAAGGUCAUTT-3′.

### Xenograft Mouse Model

Four-week-old Balb/c female nude mice were obtained from Charles River Laboratory (Beijing, China). A total of 1 × 10^7^ cells/0.1 mL in PBS were injected subcutaneously into the right hind limb of each mouse. The tumor size was measured every other day, and tumor volume was calculated using the following formula: volume (mm^3^) = a^2^ × b/2, where a = length (mm) and b = width (mm).

### Immunohistochemical (IHC) Staining

Tumors and organs were stained with H&E and examined using a NIKON ECLIPSE E100 microscope. IHC staining for PAI-1 and cystatin D was performed in tumors and imaged by NIKON DS-U3.

### Statistical Analysis

All statistical analyses were performed using GraphPad Prism 7. Quantitative data were expressed as means ± standard deviation (SD). The differences between two groups were analyzed using Student’s *t*-test, while those between multiple groups were analyzed using analysis of variance (ANOVA) followed by Tukey as the *post hoc* test. All data were derived from three independent experiments, and differences were considered significant at *P* < 0.05.

## Results

### VD_3_ Enhanced Radiosensitivity of CRC Cells *in vitro*

**FIGURE 1:**
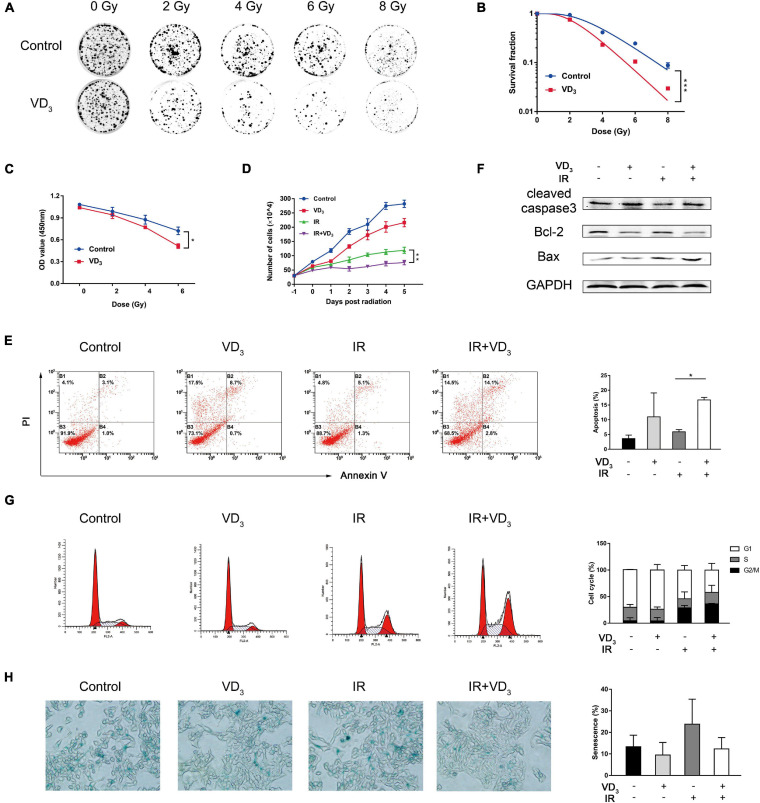
VD_3_ enhanced radiosensitivity of CRC cells *in vitro.* **(A,B)** Clonogenic assays were used to determine the radiosensitization effects of VD_3_ on SW480 cells. Cells were pretreated with VD_3_ (100 nM) and subsequently with the indicated IR doses (0, 2, 4, 6, and 8 Gy). Number of colonies was calculated after 2 weeks. **(C)** Cell proliferation evaluated by the CCK8 assay showed that IR plus VD_3_ suppressed cell growth in a IR dose-dependent manner. **(D)** Combination treatment suppressed cell growth in a time-dependent manner. Cell numbers were recorded every day. **(E)** Apoptosis analysis of VD_3_ or IR treated SW480 cells. Cells were pretreated with VD_3_ (100 nM), or IR (6 Gy) and then analyzed by Annexin V/PI. **(F)** Expression of apoptosis proteins were assessed by western blot. **(G)** Cell cycle analysis of VD_3_ or IR treated SW480 cells. Cells treated with the indicated treatments were stained with PI and then analyzed by flow cytometry. **(H)** Cellular senescence was detected by SA-β-gal staining. *p < 0.05, **p < 0.01, ***p < 0.001.

VDR (vitamin D receptor) was evident both in SW480 and HCT116 cell lines (Supplementary Figure 1A), which was considered to be crucial for vitamin D to function in the genomic pathway. After detecting IC50 of VD_3_ (Supplementary Figure 1B), we determined 20% of IC50 as the sensitizer dose for subsequent experiments, which was consistent with the dose concentration (100 nM) in previous studies (Bristol et al., 2012; Sharma et al., 2014). Next, we explored the colony-formation abilities of SW480 cells treated with VD_3_ and IR (0, 2, 4, 6, and 8 Gy), which demonstrated that VD_3_ reduced colonies in the dose-dependent way (Figures 1A,B). Clonogenic survival of HCT116 has been provided in Supplementary Figures 1C,D. Moreover, CCK8 assay was consistent with the colony formation results (Figure 1C and Supplementary Figure 1E). According to the above results, 6 Gy was selected for subsequent IR scheme. Next, trypan blue assay revealed that VD_3_ prohibited viability of irradiated cells in a time-dependent manner, the significant difference between IR alone and combination group appeared at least 24 h post IR (Figure 1D and Supplementary Figure 1F). Hence, 24 h post IR was selected as the time point for observing radiosensitizing effect. Given that vitamin D plays an essential role in induction of apoptosis (Polar et al., 2003; DeMasters et al., 2004), we performed apoptosis assay and found that there were more apoptotic cells with combined treatment compared to IR alone (Figure 1E). In parallel with the flow cytometry results, the expression of cleaved-caspase 3 and Bax was increased with the decrease of Bcl-2 (Figure 1F). Additionally, cell cycle distribution was examined to find that G2/M phase arrest was remarkably induced by IR, but no significant difference was found between IR alone and the combination group (Figure 1G and Supplementary Figure 1G). Besides, more senescent cells were detected in response to IR and there was no difference in the presence or absence of VD_3_ (Figure 1H). Therefore, VD_3_ displayed elevated sensitivity of CRC cells to IR and the underlying mechanisms remained to be established although apoptosis appeared to partially interfere with the cell survival.

### VD_3_ Combined With IR Participated in DNA Damage and Autophagy

**FIGURE 2:**
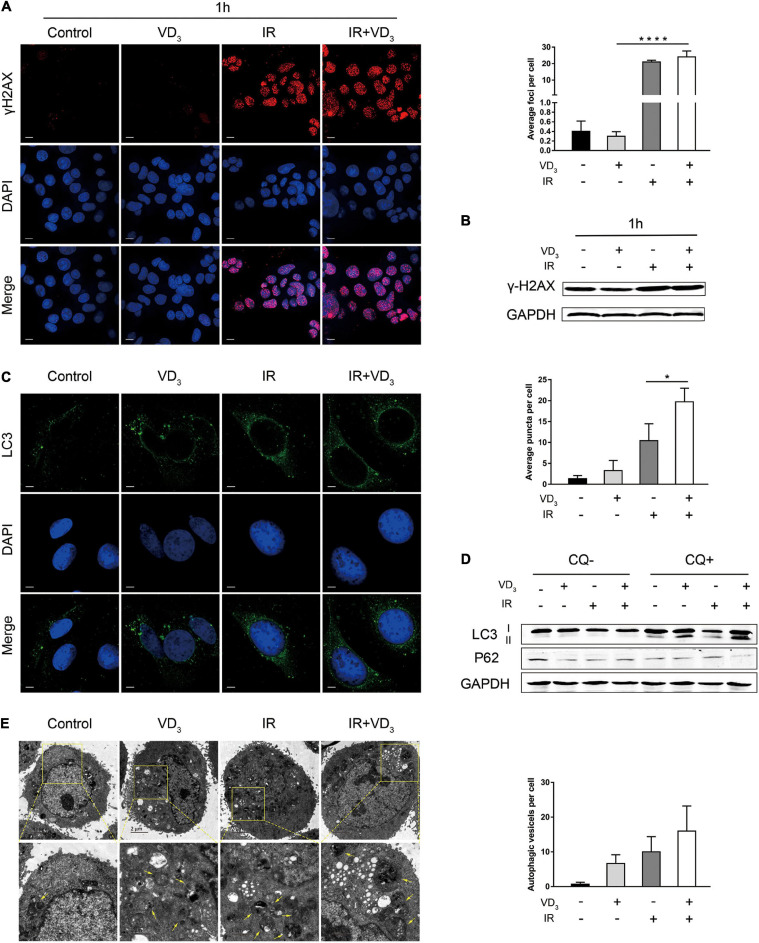
VD_3_ combined with IR participated in DNA damage and autophagy. **(A)** SW480 cells were treated with Control, VD_3_, IR, and IR plus VD_3_ treatment for 1 h post IR. Immunofluorescence staining for γ-H2AX foci formation (DNA damage marker). Scale bar: 20 μm. **(B)** γ-H2AX expression was detected by western blot. **(C)** Representative confocal fluorescence of LC3B puncta (autophagy marker) after the indicated treatments. Scale bar: 20 μm. **(D)** LC3B expression was analyzed by western blot. **(E)** Representative transmission electron microscope (TEM) images with indicated treatments in SW480 cells. Scale bar: 1μm. Yellow arrowheads denote autophagic vesicles. Autophagic vesicles per cell were quantified. *p < 0.05, ****p < 0.0001.

IR is known to induce double-strand breaks (DSBs) by directly damaging DNA, and vitamin D was also reported to have influence on DNA damage (Tremezaygues et al., 2010; Gonzalo, 2014), so we hypothesize that VD_3_ might affect radiosensitivity in the DSB-dependent manner. Immunofluorescence demonstrated that γ-H2AX foci in the nuclei (key events in DSBs) appeared 1 h after IR (Figure 2A), which sustained for 24 h (Supplementary Figure 2A). In addition, γ-H2AX expression was also detected by western blot (Figure 2B and Supplementary Figure 2B). As presented above, IR induced DSBs remarkably, but neither the foci nor the protein of γ-H2AX was affected by VD_3_. Given that autophagy has been documented to be associated with vitamin D mediated sensitivity (Wilson et al., 2011), we assessed the quantity of LC3B puncta in tumor cells with combination treatment. As shown in Figure 2C, a greater extent of LC3B puncta was detected in the combined treatment than that with either treatment alone. In accordance with this tendency, western blot revealed that more LC3B-I was converted to LC3B-II in combination group with addition of chloroquine (CQ, autophagy inhibitor), accompanied with obvious p62 degradation (Figure 2D). This induction of autophagy was further confirmed by transmission electron microscopy where autophagosome formation was mildly elevated with IR plus VD_3_ (Figure 2E). Overall, although the antitumor effects of combination treatment involved DNA damage and autophagy, minor differences could not explain the additive antitumor effectiveness induced by vitamin D in CRC.

### VD_3_ Inhibited EMT in Combination With IR

**FIGURE 3:**
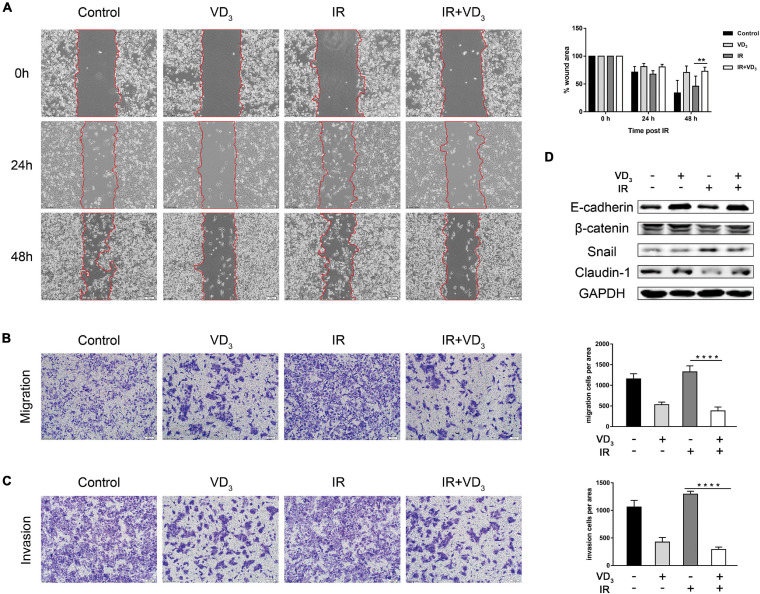
VD_3_ inhibited EMT in combination with IR. **(A)** Cell migration rates of the four groups was measured by the wound-healing assay. Images of wound closure were taken at 0, 24, and 48 h after scratching the cell layer. Scale bar: 100 μm. **(B,C)** Migration and invasion of treated cells were evaluated by transwell assays. Scale bar: 100 μm. Cellular migration and invasion were plotted as the average number of cells in three different fields. **(D)** Representative expression of EMT markers determined by western blot. **p < 0.01, ****p < 0.0001.

EMT is a highly dynamic process which is able to impart cells with several traits like tumor-initiating properties, increased motility and invasive capacity, including the resistance to several treatment strategies (Dongre and Weinberg, 2019; Yang et al., 2020). To determine whether vitamin D is involved in regulating cells capable of invading and metastasizing, we performed wound healing and transwell assays. As observed in SW480 cells, IR plus VD_3_ significantly inhibited the migration rate in comparison to IR alone (Figure 3A), and transwell migration assay also displayed a dramatic reduction in migration with the combined treatment (Figure 3B), as well as a greater loss of tumor invasiveness (Figure 3C). Meanwhile, upregulation of epithelial markers (E-cadherin, Claudin-1) and downregulation of mesenchymal marker (Snail) were determined by western blot (Figure 3D). The above findings are all associated with antitumor effects, and the mechanisms by which tumor cells respond to therapeutics are considerably more complex. RT in combination with vitamin D can simultaneously initiate different types of cell death. How vitamin D could confer radiosensitivity by regulating phenotypes involved in cell death remains to be established in greater detail.

### Proteomics Revealed Influence on EMT

**FIGURE 4:**
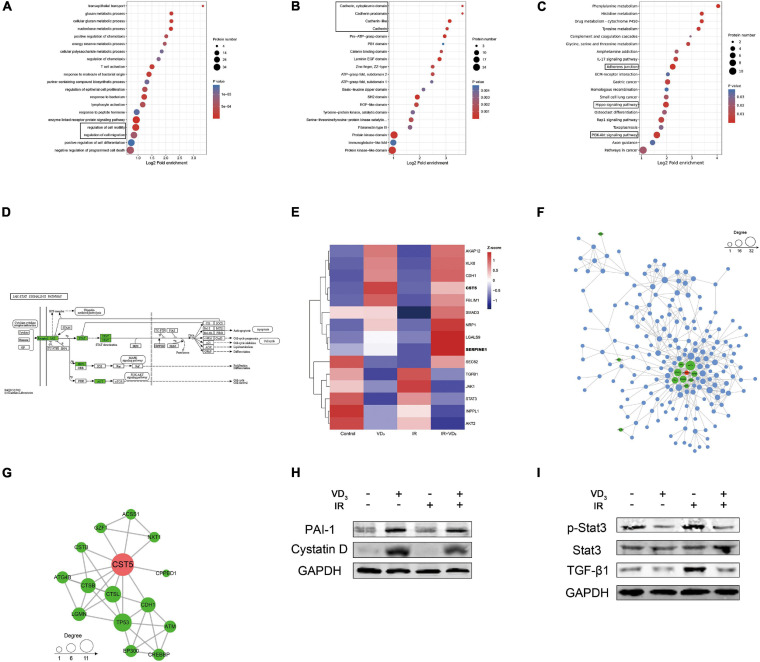
Proteomics revealed influence on EMT. **(A–C)** Functional enrichment analysis of differentially expressed proteins based on Gene Ontology (GO) terms. The circle size indicates the number of enriched genes in each term. Each color indicates a different *p*-value as indicated. The fold enrichment represents the ratio of enriched proteins in the selected term to the total number of proteins in the term. **(D)** The Kyoto Encyclopedia of Genes and Genomes (KEGG) pathway analysis of differentially expressed proteins identified by proteomics. **(E)** Hierarchical clustering analysis of representative proteins associated with EMT, relative increase or decrease in expression was shown by indicated colors. **(F,G)** Protein-protein interaction (PPI) network among the heatmap proteins. **(H)** Western blot validation of the changes in cystatin D and PAI-1 protein expression with indicated treatment. **(I)** Immunoblot analysis of the changes in JAK/STAT3 and TGF-β1/Smad3 signaling pathway protein expression with indicated treatment.

To further explore the involved molecular mechanisms by which vitamin D exerted its anticancer action, we conducted quantitative proteomics of SW480 cells pretreated with VD_3_ or IR. Gene Ontology (GO) for molecular function (MF) revealed that differentially expressed proteins (DEPs) were significantly enriched in the terms of “regulation of cell migration” and “regulation of cell motility” (Figure 4A). Protein domain analysis of DEPs was significantly enriched in “Cadherin” and “Cadherin like” terms (Figure 4B). The Kyoto Encyclopedia of Genes and Genomes (KEGG) pathway analysis showed terms such as “Adherents junction” and “Hippo signaling” associated with EMT were significantly enriched (Figure 4C). Further pathway analysis revealed the significant influence on “JAK/STAT3 signaling pathway” and “TGF-β/Smad3 signaling pathway” (Figure 4D and Supplementary Figure 3A). Moreover, the heatmap of top 15 DEPs were mostly involved in EMT (Figure 4E), particularly with upregulation of cystatin D (*CST5*) and PAI-1 (*SERPINE1*) in the combination group. Specifically, proteins associated with these two candidates were further determined by protein-protein interaction (PPI) network analysis (Figures 4F,G). Based on the previous studies of cystatin D and PAI-1 (Alvarez-Diaz et al., 2009; Mahmood et al., 2018; Breznik et al., 2019), we determined to investigate if cystatin D and PAI-1 were able to regulate radiosensitivity via EMT modulation. This hypothesis can be potentiated by protein validation, as shown in Figure 4H where the expression of cystatin D and PAI-1 were remarkably upregulated in case of combined therapy. Similarly, proteins in signaling pathways associated with EMT were also validated by western blot, the results were consistent with GO and KEGG analysis (Figure 4I and Supplementary Figure 3B). More recently, EMT is reported to confer therapy resistance by eliciting stem cell maintenance (Lambert and Weinberg, 2021). Interestingly, we indeed observed that colorectal cancer stem cells co-treated with VD_3_ and IR developed fewer and smaller spheroids (Supplementary Figure 3C).

### Gene Inhibition Partially Reversed Sensitivity to IR by Regulating EMT

**FIGURE 5:**
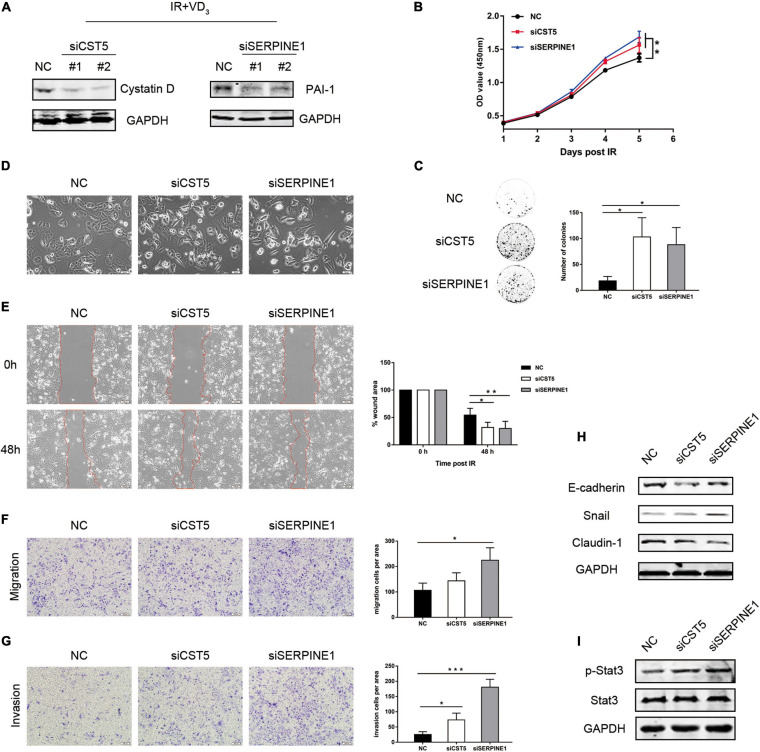
Gene inhibition partially reversed sensitivity to IR by regulating EMT. **(A)** SW480 cells transfected with the indicated small interfering RNAs (siRNAs) were treated with the combination treatment (IR + VD_3_) and analyzed by western blot. **(B)** siRNAs of *CST5* and *SERPINE1* promoted cell viability, which was recorded everyday using CCK8. **(C)** *CST5* and *SERPINE1* loss enhanced colony formation. Colonies were detected after incubating for 14 days. **(D)** Morphological changes following siRNAs treatment. **(E)** Cell migration rate was compared by the wound-healing assay. Observations were recorded at 0 and 48 h after scratching the cell layer. Scale bar: 100 μm. **(F,G)** Transwell assay detected the effect of siRNAs on cellular migration and invasion. Scale bar: 100 μm. **(H)** Expression of EMT markers were examined by western blot. **(I)** Expression of JAK/STAT3 signaling proteins were examined by western blot. *p < 0.05, **p < 0.01, ***p < 0.001.

Considering the possibility that cystatin D and PAI-1 might function in promoting sensitivity to IR via the reversal effects of vitamin D in EMT, colony formation and transwell assays were performed. The effects of knockdown on *CST5* and *SERPINE1* were validated at the protein level (Figure 5A). As observed in Figure 5B, *CST5* and *SERPINE1* silencing partially reversed the synergistic antitumor effectiveness induced by VD_3_. Similarly, the colonies of siRNAs were much more than that in the NC group (Figure 5C). Moreover, reversal effect of cystatin D and PAI-1 in EMT was firstly observed in morphological changes. Compared with the NC group, the siRNA cells became sharper and adopted a spindle-shaped mesenchymal phenotype (Figure 5D). Furthermore, inhibition of *CST5* and *SERPINE1* greatly increased cell metastatic powers, especially for knockdown of *SERPINE1* (Figures 5E–G). Similarly, immunoblotting analysis demonstrated that siRNAs downregulated the expression of epithelial marker E-cadherin and Claudin-1 but upregulated expression of EMT-TFs Snail (Figure 5H). Moreover, as demonstrated by proteomics, gene inhibition showed a significant downregulation of p-Stat3 (Figure 5I). These results showed that treatment with knockdown on *CST5* and *SERPINE1* reverted EMT inhibition effect of VD_3_ and restored resistance to IR, probably by counteracting JAK/STAT3 signaling, which is typically induced during EMT and appears to confer therapy resistance by regulating genes involved in cell proliferation (Jin, 2020).

### VD_3_ Enhanced Sensitivity to IR *in vivo* Without Additional Toxicity

**FIGURE 6:**
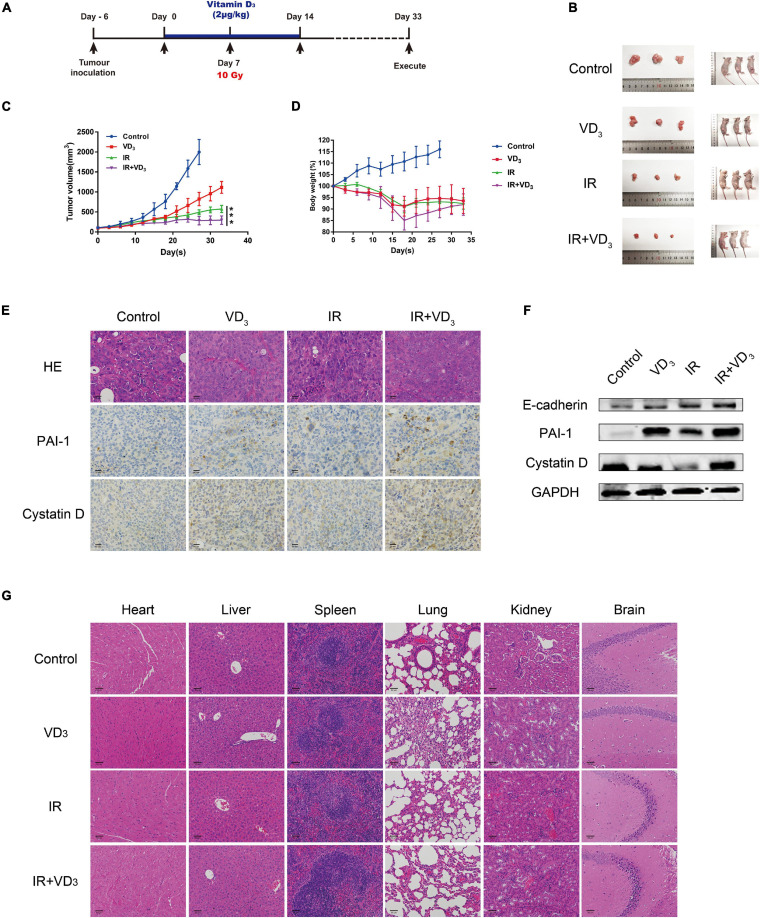
VD_3_ enhanced sensitivity to IR *in vivo* without additional toxicity. **(A)** Experimental design was shown. Day -7: SW480 cells (1 × 10^7^) were subcutaneously inoculated into the right hind limb of nude mice; Day 0: Tumors were pretreated with VD_3_ (2 μg/kg, intraperitoneal injection); Day 7: Tumors were irradiated once with 10Gy. Tumor size was measured every other day. **(B)** Images of the dissected tumors. Tumors in the control group were harvested on day 28, and rest groups were harvested on day 33. **(C,D)** Growth curves for xenograft tumors and body weight of nude mice. **(E)** IHC of cystatin D and PAI-1 and H&E staining in xenograft tumors. Scale bar: 20 μm. **(F)** Protein levels of cystatin D and PAI-1 in tumors were tested by western blot. **(G)** Images of H&E staining for organs from the mice. Scale bar: 50 μm. ***p < 0.001.

The antiproliferative additive effect of VD_3_ was further evaluated *in vivo*, experiment scheme was depicted in Figure 6A. In line with the findings *in vitro*, VD_3_ acted in combination with IR by achieving a further reduction of tumor growth when compared to that with the IR treatment alone, with no significant alteration in body weight (Figures 6B–D). As shown in IHC and western blot analysis, a significant elevation of cystatin D, PAI-1, and E-cadherin suggested a substantial EMT-associated pathway in vitamin D mediated sensitization to IR (Figures 6E,F), which were consistent with the outcome *in vitro*. In particular, VD_3_ has been shown to delay tumor progression without increasing the toxicity, no notable morphologic tissue change was observed in all groups (Figure 6G). These results confirmed that vitamin D as potential radiosensitizer has been validated in CRC xenograft models, the co-treatment with VD_3_ and IR was able to elicit a synergistic antiproliferative effect without causing side effects.

**FIGURE 7:**
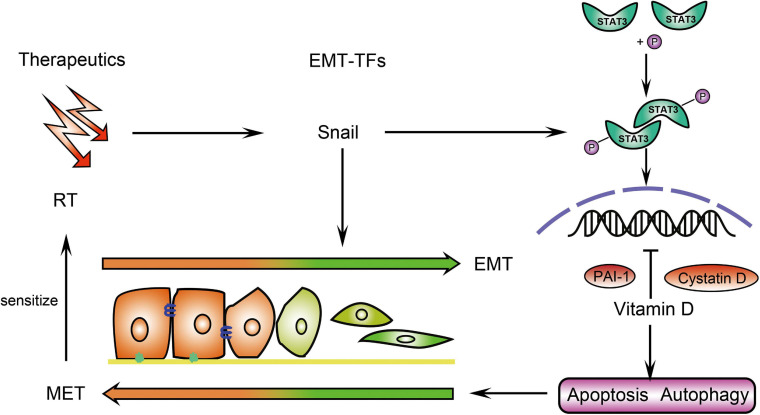
Schematic illustration of how vitamin D promotes the radiation sensitivity of CRC. In CRC cells, vitamin D promoted cystatin D and PAI-1 to inhibit p-Stat3 expression, which was induced by EMT-inducing transcriptions factors (EMT-TFs) Snail and thus contributing to EMT. The induction of apoptosis and autophagy by vitamin D ultimately increased the sensitivity of the tumor to radiotherapy (RT).

In conclusion, EMT was the key mechanism responsible for the sensitivity effect of vitamin D on IR in CRC. Vitamin D inhibited EMT via induction of cystatin D and PAI-1, which enhanced the radiation therapeutic effects on CRC (Figure 7).

## Discussion

Our study showed that the treatment with vitamin D restored sensitivity to IR validated by various phenotypes. Although apoptosis, autophagy, and EMT were all engaged in the machinery of combination treatment, influence on EMT was most significant and results of proteomics led to the notion that EMT should be determinant for this regulation. EMT is well known to facilitate tumor metastasis, a series of assays for assessing migration and invasion ability was first performed. Subsequently, by detecting proliferation assays such as colony formation and CCK8, we evaluated the influence of combined therapy on CRC ability to demonstrate that CRC ability was an enhanced proliferation activity, which could be deregulated by vitamin D, and notably involved in treatment response upon inhibition of EMT, thus confirming vitamin D involvement in cancer growth control.

Cells undergoing EMT not only behave invasive and motile but also acquire resistance to therapeutic agents. Therapeutic efficacy will be attenuated if tumor migratory and invasive properties are activated. Increasing evidence supports that EMT can indirectly influence radioresistance by regulating genes involved in cell death. ZEB1 was reported to promote DNA damage repair (Zhang et al., 2014). Moreover, EMT-TFs can promote therapy resistance by antagonizing p53-mediated apoptosis (Wu et al., 2005; Kurrey et al., 2009). EMT-TFs can also confer resistance to oxaliplatin-based and cisplatin-based chemotherapies in several cancers (Guo et al., 2012; Soyoung et al., 2013). In addition to conferring resistance to chemotherapeutic regimens, it has been recently reported that EMT is also associated with a refractory response to immunotherapy (Dongre et al., 2017). When EMT is elicited in carcinoma cells, the resulting quasi-mesenchymal neoplastic cells can modify the stromal signals and influence the immune response. There is study demonstrated that EMT-induced immunosuppressive effects can be reversed by abrogation of EMT-TFs (Kudo-Saito et al., 2009). These data establish EMT as a sensitization switch that repression of EMT-TFs can restore sensitivity of carcinomas to therapeutic agents. Several signaling pathways are associated with EMT, including the Wnt/β-catenin, PI3K/AKT/NF-κB, TGF-β1/Smads, and JAK/STAT3 pathways (Dongre and Weinberg, 2019). Among these pathways, JAK/STAT3 plays a classic role in promoting tumor invasion and metastasis (Jin, 2020). In this study, JAK/STAT3 pathway interfered with treatment response by regulating EMT, which confers on cancer cells a greater resistance to elimination by therapeutics.

EMT can also give rise to a variety of quasi-mesenchymal cell states, which can function as cancer stem cells (CSCs) with elevated tumor-initiating potential and display elevated resistance to several therapeutic regimens compared with non-CSCs (Lambert and Weinberg, 2021). EMT can directly induce stem-cell properties in epithelial cells (Mani et al., 2008), including the upregulation of stem cell marker CD44 and elevated spheroid formation (Polyak and Weinberg, 2009). Moreover, properties like self-renewal and tumorigenicity associated with the stem-like phenotype were conferred to CRC cells when Snail was activated (Hwang et al., 2011). Recent data has revealed that vitamin D analog BXL0124 decreased the CD44 level (So et al., 2011). Additionally, there was evidence that calcitriol could directly influence tumor-initiating cells (also known as CSCs) by reducing spheroid formation (Jeong et al., 2015). Nonetheless, whether VD_3_ can influence treatment response by regulating CSCs in CRC merits further investigation.

Based on the proteomics results, our efforts were focused on exploring the function of cystatin D and PAI-1. Cystatin D, a member of cystatin family, is found to exert anti-migratory effects by suppressing the *c-myc* and transcriptional activity of β-catenin as well as induction of E-cadherin (Alvarez-Diaz et al., 2009). Furthermore, a study indicated that cystatin D level could be elevated with combining calcitriol treatment and p53 activation (Huenten and Hermeking, 2015). Taken together, our results of cystatin D were consistent with the previous findings. Urokinase-type plasminogen activator (uPA) with its receptor (uPAR) displays crucial role in tumor progression and metastasis, and PAI-1 is the predominant endogenous inhibitor of uPA (Thorsen et al., 1988). uPA/PAI-1 ratio has been reported to be associated with the invasive behavior in oral squamous cell carcinomas (Hundsdorfer et al., 2005). Conversely, it was also found that PAI-1 could suppress tumor migration by interacting with the binding site between vitronectin and integrin (Stefansson and Lawrence, 1996). It was further documented that PAI-1 promoted apoptosis in prostate cancer (Chen et al., 2008). Given the contradictory evidence, the role of PAI-1 in tumor aggressiveness is complex, it can potentially mitigate or enhance cancer progression in the context of different neoplasia. In our study, inhibition of PAI-1 was found to activate excessive proliferation and invasive activity in SW480 cells, which demonstrated the essential role of PAI-1 in VD_3_ mediated radiation sensitization. However, the correlation between PAI-1 and JAK/STAT3 signaling remains to be investigated in greater detail.

Taken into consideration the available data in our study, it can be concluded that vitamin D can act on sensitizing IR at different levels. The studied phenotypes involve EMT inhibition and induction of apoptosis and autophagy. Moreover, the role of cystatin D and PAI-1 on modulation of EMT could be a promising field for future investigation. As for the clinical application, further studies should focus on hypercalcemia induced by supraphysiological concentrations of vitamin D, although structural analogs of vitamin D are being developed. Moreover, it will be of great interest to determine when vitamin D should be introduced (the optimal time point) and how long vitamin D will perform during the treatment process (the total therapeutic dose) to make better decision for vitamin D-adjuvant therapy.

## Data Availability Statement

The original contributions presented in the study are included in the article/Supplementary Material, further inquiries can be directed to the corresponding author/s.

## Ethics Statement

The animal study was reviewed and approved by the Laboratory Animal Ethics and Welfare Committee, School of Basic Medicine, Jilin University.

## Author Contributions

XY and GC conceived, designed the experiments, and wrote the manuscript. XY, QW, and BL performed the experiments. XY and QW analyzed the data. NZ and BL contributed to material and analysis tools. All authors read and approved the final manuscript.

## Conflict of Interest

The authors declare that the research was conducted in the absence of any commercial or financial relationships that could be construed as a potential conflict of interest.

## Publisher’s Note

All claims expressed in this article are solely those of the authors and do not necessarily represent those of their affiliated organizations, or those of the publisher, the editors and the reviewers. Any product that may be evaluated in this article, or claim that may be made by its manufacturer, is not guaranteed or endorsed by the publisher.
