# Supplementary material for: Vitamin D Enhances Radiosensitivity of Colorectal Cancer by Reversing Epithelial-Mesenchymal Transition
Source: Front Cell Dev Biol. 2021 Aug 4;9:684855. doi: 10.3389/fcell.2021.684855 (PMC8371408; doi:10.3389/fcell.2021.684855)
Supplement: Supplementary file 1 [file Data_Sheet_1.PDF]

Supplementary Fig. 1.

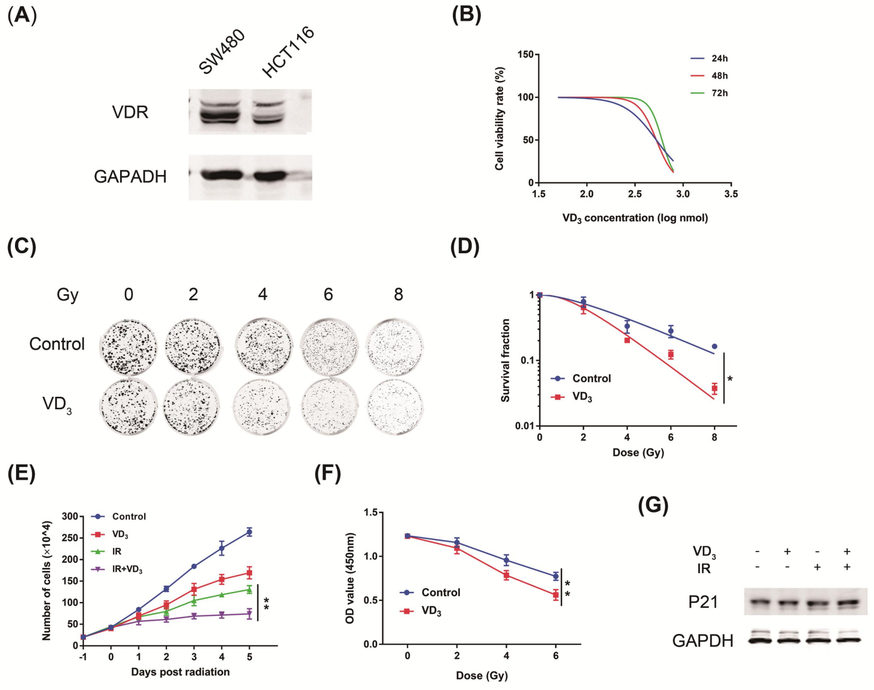

VD_3 enhanced radiosensitivity of HCT116 in vitro

A, VDR was evident in SW480 and HCT116 cell lines. **B**, IC50 of VD_3 on SW480 cells determined by CCK8. **C and D**, Clonogenic assays were used to determine the radiosensitization effects of VD_3 on HCT116 cells. **E**, Cell proliferation inhibition of VD_3 was evaluated in HCT116 cells. **F**, Trypan blue assay detected cell viability in treated HCT116 cells. **G**, p21 (Cell cycle protein) determined by western blot. $*p < 0.05$, $**p < 0.01$, $***p < 0.001$, $****p < 0.0001$.

Supplementary Fig. 2.

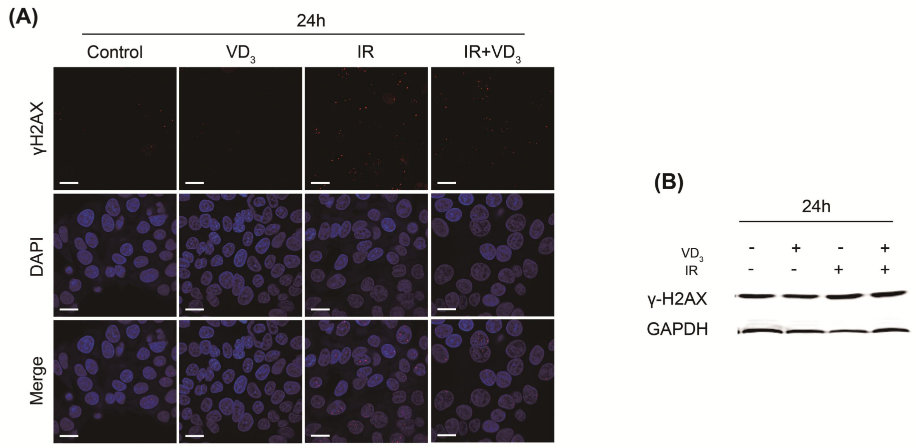

VD_3 combined with IR participated in DNA damage

A, γ-H2AX foci formation for 24 h post IR in SW480 cells. Scale bar: 20 μm. **B**, γ-H2AX expression for 24 h post IR was detected by western blot.

Supplementary Fig. 3.

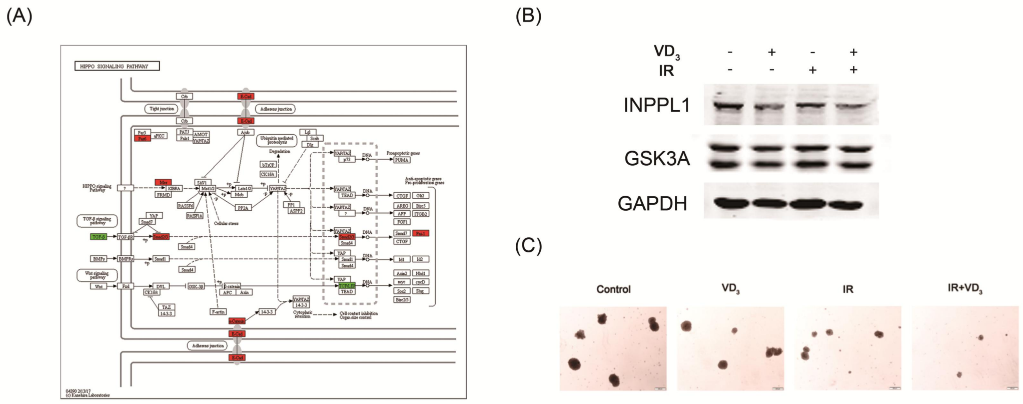

Proteomics revealed influence on EMT

A, KEGG pathway analysis of differentially expressed proteins enriched in

TGF-β/Smad3 signaling. **B**, Immunoblot analysis of the downregulated proteins determined in proteomics. **C,** Sphere formation of colorectal cancer stem cells with combined treatment. Scale bar: 200μm.
